# Assessment of Pollen Limitation and Pollinators’ Contribution in Soybean (*Glycine max*)

**DOI:** 10.3390/plants14192964

**Published:** 2025-09-24

**Authors:** Silvio Eugenio Castillo, Roxana Aragón, Natacha Chacoff

**Affiliations:** 1 Instituto de Ecología Regional (IER), Universidad Nacional de Tucumán (UNT)-Consejo Nacional de Investigaciones Científicas y Técnicas (CONICET), Yerba Buena 4107, Argentina; roxaragon@gmail.com (R.A.); nchacoff@gmail.com (N.C.); 2 Facultad de Ciencias Naturales e Instituto Miguel Lillo, Universidad Nacional de Tucumán, San Miguel de Tucumán 4000, Argentina

**Keywords:** Soybean (*Glycine max*), hand pollination, pollen limitation, pollinator contribution, experimental pollination

## Abstract

Soybean (*Glycine max*) is a predominantly self-pollinating crop; however, its flowers exhibit traits associated with insect pollination. While several studies report yield benefits from floral visitation, others suggest little or no effect, and few have assessed pollen limitation through direct hand-pollination experiments. Here, we assess pollinator contribution and pollen limitation through two manipulative common garden experiments using different soybean cultivars. First, we assessed the contribution of pollinators by comparing reproductive variables between caged (pollinator excluded) and open-pollinated plants over two growing seasons. Second, we supplemented flowers with cross-pollen to test for pollen limitation, evaluating pollen-tube growth, pod set, seed number per pod, and seed weight. Pollinator exclusion did not significantly reduce total pod or seed production per plant, but open pollination increased seed set (seeds per flower) by ~16%. In contrast, hand supplementation substantially improved reproductive success at the flower level, tripling pod set probability and increasing seed number per pod by 40%. Additionally, both open-pollinated and hand-pollinated flowers exhibited higher pollen-tube growth relative to autonomous selfing. These findings highlight that even in largely self-compatible crops like soybean, additional pollen input can enhance reproductive success and help bridge the gap between the ecological and agronomic dimensions of pollination.

## 1. Introduction

Pollination, the transfer of pollen from anthers to stigmas, is vital to the reproduction of most flowering plants. Approximately 90% of angiosperm species worldwide rely on animals—primarily insects—to mediate pollination, ensuring their sexual reproduction, which is critical for ecosystem functioning [1,2]. In agriculture, pollinators significantly enhance yields or fruit quality in many crops [3,4]. However, the magnitude of pollinator impact on crops varies greatly, from species that rely almost entirely on insect pollination (dioecious and obligate outcrossers) to self-compatible crops that generally self-pollinate but can also show marginal, and sometimes substantial, yield gains from insect visits [3,4,5].

Soybean (*Glycine max* (L.) Merrill) is an annual legume widely cultivated for its protein-rich seeds and oil, forming the basis of myriad products for human consumption and livestock feed, and is a key global commodity [6]. Although the flowers are hermaphroditic and largely autogamous, soybean exhibits morphological traits that promote pollination by insects, such as nectar guides, floral nectaries, and a specialized papilionaceous structure typical of the Fabaceae [7].

In Argentina, soybean cultivation covers extensive areas and is a major driver of land-use change. This phenomenon is particularly pronounced in the semi-arid Chaco region, which has some of the highest deforestation rates worldwide [8,9]. Novel cultivars and transgenic traits (e.g., glyphosate tolerance and Bt insect resistance) have extended the agricultural frontier into previously marginal lands, often displacing native habitats, other crops, and grasslands used for livestock production [10]. Deforestation diminishes habitat for pollinators and other beneficial arthropods, potentially weakening ecosystem services critical for sustainable crop production [11,12]. Understanding the extent to which soybean relies on animal pollination, and the degree to which pollen availability can limit productivity can provide essential insights for making informed decisions on habitat conservation and pollinator management, crucial for implementing ecological intensification strategies [13,14,15].

While several studies have demonstrated that biotic pollination can enhance soybean yield [16,17], others have found negligible effects, even under similar pollinator exclusion protocols [18,19]. These inconsistencies highlight how pollinator contribution may vary depending on cultivar traits, local environmental conditions, and the identity or abundance of floral visitors [20,21,22]. In the global synthesis by Klein et al. (2007), soybean was classified as having modest pollinator dependence (10–40%), and more recently Siopa et al. (2024) refined this estimate, reporting cultivar-level variability ranging from 0 to 37% (mean ~19%) [3,4]. Importantly, these estimates are derived solely from open-pollination versus exclusion experiments; no studies have tested the effect of hand-supplementation on seed or pod production. This omission may bias pollinator dependence estimates by overlooking potential pollen limitation [4,23]. To our knowledge, only Evans et al. (2023), working with vegetable soybean (edamame), evaluated the benefits of cross-pollination through direct hand supplementation, reporting significant gains in fruit weight and marketable yield [24]. Thus, we still lack direct experimental evidence in grain soybean to determine whether reproduction is limited by pollen availability or quality. Because the absence of pollen limitation data in pollinator-dependent crops has been shown to significantly underestimate pollinators’ potential contribution to production [4], addressing this gap is crucial to clarify the role of pollinators in soybean production and to inform sustainable crop management under changing agroecosystem conditions.

In this research, we examined soybean pollination biology in two manipulative common garden experiments. First, we tested pollinator contribution in soybeans by comparing plants exposed to natural pollination with plants enclosed in cages (i.e., excluded from pollinators) during flowering, across two growing seasons. Second, we assessed pollen limitation by performing manual cross-pollinations to supplement pollen, thereby observing potential gains in pollen tube growth, pod set probability, seeds per pod, and seed weight. Our findings help clarify the mechanisms involved in soybean seed production and highlight the potential contribution of pollinators to improving yields.

## 2. Materials and Methods

### 2.1. Experiment Location

This study was conducted in the common garden experimental area of the Instituto de Ecología Regional (26°46′38.15″ S, 65°19′30.64″ W; 710 m asl), located within the Parque Sierra de San Javier protected area. The site lies at the upper boundary of the Pedemontane Forest of the Yungas ecoregion within proximity of secondary woodland patches. Most of the nearby areas are dominated by secondary vegetation, with *Ocotea porphyria* and *Blepharocalyx salicifolius* in the canopy stratum, *Allophyllus edulis* and *Eugenia uniflora* in the subcanopy, and the shrub *Psychotria carthagenensis* dominating the understory [25]. Several epiphytic species and herbs are also present. The flowering period of most of these species occurs during spring and early summer (September–November), and some, including *Cupania vernalis*, *Solanum riparium*, *Cestrum parqui*, and *Vassobia breviflora*, may overlap with soybean flowering, potentially attracting shared pollinators. The climate is subtropical with a pronounced rainy season (November–March). Daily temperatures in the region typically range from 18 to 35 °C during the summer months [26]. The presence of nearby forest fragments provides potential for pollinator activity.

### 2.2. Experiment Setup

We conducted pollinator exclusion experiments from January to May over two consecutive growing seasons (2017 and 2018) and performed a hand-pollination supplementation experiment only in 2018. Experiments used potted soybean plants placed outdoors in full sunlight. Two soybean seeds of the same cultivar were sown in 10 L pots in early January, coinciding with the typical sowing period in the field. After germination, we thinned each pot to one seedling once the V3 stage was reached in late January [27]. Pollinator exclusion cages were installed in mid-February, just prior to the onset of flowering, and removed at the end of the flowering period in late March. Plants were watered regularly to avoid water stress, and a commercial systemic insecticide, GlacoXAN D-sist^®^ (Glacoxan, Buenos Aires, Argentina), was applied weekly during stages R4–R6, from mid-March to mid-April, to minimize herbivore damage. Harvest of all mature pods and seeds per plant began in the last days of April and continued into the first days of May in both seasons. A timeline figure summarizing sowing, cage setup, flowering onset, insecticide applications, and harvest times can be found in the Supplementary Material (Figure S1).

We selected four commercial soybean cultivars commonly grown in the semi-arid Chaco of Northwest Argentina. Three (‘A8000’, ‘DM8002’, and ‘NS7211’) carry a glyphosate-tolerance trait. The fourth (‘DM8277’) combines an enhanced glyphosate-tolerance trait with a *Bacillus thuringiensis* (Bt) trait conferring resistance to lepidopteran pests; this combination is marketed as INTACTA RR2 PRO [28]. In addition to their transgenic status, these cultivars differ in floral morphology and growth habit: ‘A8000’ and ‘DM8002’ have purple flowers, while ‘NS7211’ and ‘DM8277’ have white flowers; ‘DM8277’ is an indeterminate cultivar, whereas the other three are determinate, referring to differences in growth habit, specifically whether the main stem continues to elongate after flowering begins. All belong to late maturity groups (MG VII–VIII). Seeds were obtained from the Estación Experimental Obispo Colombres (a research institution that establishes the field productive characteristics of different cultivars).

### 2.3. Pollinators’ Contribution

To assess the contribution of pollinators to soybean, we tested whether different reproductive variables change when pollinators are excluded during flowering. Two treatments were applied: (1) Enclosed, where plants were placed under nylon-mesh cages (1.5 m × 0.5 m × 1.0 m) with 1.5 mm mesh size to block insect entry, and (2) Open, where plants were accessible to all pollinators. Cages were inspected regularly during flowering, and no insect activity was observed inside, supporting the effectiveness of the exclusion treatment.

Each season, we randomly assigned a subset of pots from each cultivar to either Enclosed or Open conditions. In 2017, we tested five replicates (plants) per cultivar per treatment (total = 40 plants). In 2018, we used seven replicates per cultivar per treatment (total = 56 plants), giving 96 plants overall. We ensured that each cage contained a mixture of plants from multiple cultivars. Once flowering ended, the cages were removed, allowing all plants to mature under similar conditions. At harvest, all mature pods were collected from each plant, opened manually, and the number of fully developed seeds inside each pod was counted by hand; aborted or incompletely developed ovules were not included. This provided pod and seed totals per plant. To estimate plant-level seed set (seeds per flower), we quantified flower production in a subset of plants under both treatments using two approaches: direct floral censuses (2017) and indirect tallies of abscission scars (2018). Although these methods yielded different absolute flower counts, both captured a consistent treatment effect on seed set across years. Pollinator visitation was not quantified; however, we confirmed insect activity by personal observation and by the higher number of pollen tubes observed in flowers from the open treatment (see Section 3.2).

### 2.4. Pollen Limitation

To assess pollen limitation, we examined how the manual addition of pollen into flowers translates into different reproductive variables. In 2018, we selected 11 plants from three cultivars (‘A8000’, ‘DM8002’, ‘DM8277’). The cultivar ‘NS7211’ was not included due to logistical constraints of the supplementation procedure. Within each plant, some flowers were manually supplemented with cross-pollen and referred to as Supplemented Flowers (SF), while others were left unmanipulated and designated as Control Flowers (CF). SF received pollen from three to five donor plants of the same cultivar by gently exposing the stigma and rubbing anthers from the donor onto it. Although donor and recipient flowers belonged to the same cultivar, they were genetically distinct individuals, reflecting the level of genetic diversity typically found within commercial seed lots. Thus, while not representing inter-cultivar outcrossing, the supplemental pollen simulates within-cultivar pollen transfer as may occur under field conditions. All supplemented flowers were marked with one color, and control flowers were marked with a different color, allowing us to keep track of each flower’s treatment identity throughout the experiment until harvest. Across plants, the number of supplemented flowers ranged from 13 to 94 per plant (57 ± 22, n = 11), while the number of control flowers (i.e., all other flowers left for natural pollination) ranged from 41 to 118 per plant (74 ± 28). Thus, the proportion of flowers manually supplemented varied (average ~44%; range 11–69%), depending on synchronous donor availability and the logistical effort required for manual pollination. In total, we collected flower-level data from 1446 flowers (630 supplemented and 816 controls), which constituted the sampling units for the main statistical analyses. No mock-handling was applied to control flowers, so possible mechanical stimulation effects cannot be excluded. After fruit maturity, we recorded the number of pods and seeds per treatment at the flower level within each plant. As with the pollinator-exclusion experiment, pods were opened manually and only fully developed seeds were counted; aborted or incompletely developed ovules were not included in seed counts. For seed weight, all fully developed seeds from each treatment within a plant were pooled and weighed on a precision balance (0.005 g resolution). The values were then standardized to 100-seed weight. This resulted in 22 observations of seed weight at the treatment level within plants.

Pollen tube growth was assessed by collecting post-anthesis flowers from SF and CF, as well as from enclosed plants to evaluate growth under strict autonomous selfing conditions. Pollen tube staining was performed using a modified protocol based on Martin (1959) [29]. Flowers previously fixed in 70% ethanol were rinsed with distilled water and dissected under a stereomicroscope to isolate the gynoecium and a portion of the pedicel. Samples were dehydrated in 96% ethanol, and then softened by incubation in 10% NaOH for 24 h. After rinsing, the tissues were bleached in 50% sodium hypochlorite until they were cleared (~15 min), then washed three times with distilled water, and finally stained with 0.1% aniline blue. Samples were mounted on slides, and gently squashed under coverslips. Observations were made under a Nikon fluorescence microscope equipped with a high-pressure mercury lamp (Nikon Corporation, Tokyo, Japan). Pollen tubes were counted in the upper portion of the style based on callose fluorescence. Pollen tube numbers were obtained by direct microscopic counts; representative images are shown in the Supplementary Material for illustrative purposes only (Figure S2).

### 2.5. Statistical Analysis

To assess pollinator contribution, we evaluated how pollination treatment, cultivar, and seasonal factors affected reproductive variables (pods, seeds, and seed set), we ran three separate Gaussian models for (i) total pods per plant, (ii) total seeds per plant, and (iii) average seed set per plant (calculated as seeds per flower). Each model initially included three fixed effects: pollination treatment (open vs. enclosed), cultivar (‘A8000’, ‘DM8002’, ‘DM8277’, ‘NS7211’), and season (2017 vs. 2018), as well as an interaction term between treatment and cultivar. Because the season and cultivar factors had less than five levels in our dataset, we included them as fixed factors to account for their variation rather than attempting to estimate a variance component (as a random effect) with insufficient levels [30]. The seed set model was fitted using a log link function to accommodate the positive right-skewed distribution of the response variable, a common approach for ratio data in ecological studies [31]. To account for the fact that flower production was estimated using different methods in 2017 (direct census) and 2018 (abscission scars), we verified that this did not alter the inference of treatment effects on seed set by comparing model effect sizes separately for each year.

The model selection followed a stepwise process based on likelihood ratio tests (LRTs), comparing full models with reduced versions to assess the contribution of the interaction term. In all three cases, the treatment × cultivar interaction did not improve model fit (pods: *p* = 0.64; seeds: *p* > 0.05; seed set: *p* > 0.27) and was therefore excluded from the final models. For each response variable, we reported the significance of fixed effects using Wald χ^2^ tests, and assessed model explanatory power via marginal R^2^.

To assess pollen limitation, we evaluated the effects of supplemental manual cross-pollination on flower-level pod and seed production using a hurdle (two-part) generalized linear mixed model (GLMM) to account for the excess zeros in our count data. This approach allowed us to separately analyze (i) the pod set process (i.e., whether a flower produced a pod with at least one seed) and (ii) the seed production per pod, conditional on pod set.

For the zero-inflation component, we modeled the probability that a flower fails to produce a pod (i.e., a “structural zero”) using logistic regression. Fixed effects in this part included treatment (SF vs. CF), cultivar, and their interaction, with a random intercept for plant identity to account for repeated measures. Because the estimates are on the logit scale, they were converted to probabilities via the logistic transformation:

p = exp(estimate)/1 + exp(estimate),(1)

to facilitate interpretation in terms of pod set likelihood (i.e., 1 − p).

**Figure A1:**
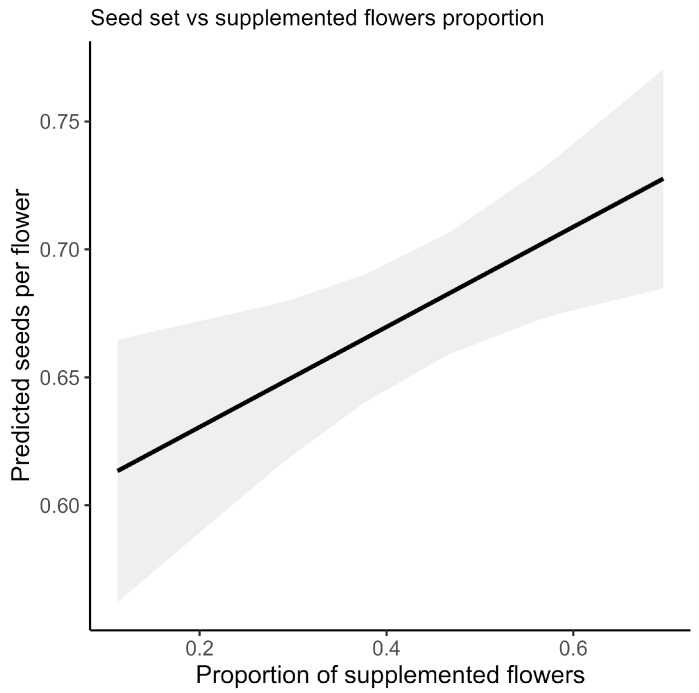
Relationship between the proportion of hand-supplemented flowers and plant-level seed set. The plot shows predicted values (±95% confidence interval) from a linear regression model.

For the conditional component, we modeled the number of seeds produced per pod (i.e., among flowers that set a pod) using a truncated Poisson distribution with a log link. The fixed effects (except for the interaction) and a random intercept for plant identity were again included. This two-part modeling strategy allowed us to disentangle the effects of supplemental cross-pollination on both the likelihood of pod set and the seed count within pods at the flower level. To explore potential within-plant trade-offs in resource allocation, we conducted an additional plant-level regression analysis relating the seed set to the proportion of supplemented flowers (see Appendix A, Figure A1).

To analyze pollen-tube growth in the style, a Poisson GLMM was initially fitted with pollination treatment (enclosed, supplemented, control), cultivar, and their interaction as fixed effects, and plant identity as a random effect. Model simplification was performed via LRTs by removing non-significant interaction and cultivar terms. Including a random intercept for plant identity improved model fit only marginally and was therefore omitted. Consequently, the final model retained only the three-level treatment factor fixed effect under a Poisson error structure.

For seed weight, a Gaussian GLMM was fitted with pollination treatment (supplemented vs. control), cultivar, and their interaction as fixed effects, and plant identity as a random intercept. Likelihood ratio tests were used to determine the relevance of the random effect and interaction. The final model retained only the fixed-effect terms.

All models were fitted in R (version 4.4.1) using the glmmTMB package [32]. The appropriate error distributions were selected by comparing candidate distributions using cumulative density plots, QQ plots, and AIC values via the fitdistrplus package [33]. Wald chi-square tests were used to test fixed effects, and model fit was evaluated using marginal and conditional pseudo-R^2^ values calculated with the MuMIn package (r.squaredGLMM; [34]). For the hurdle model, r.squaredLR was used instead.

Post hoc comparisons were conducted using estimated marginal means with the lsmeans package, applying Bonferroni corrections for multiple testing [35]. Predicted values and their confidence intervals were extracted using the ggeffects package [36] and visualized alongside raw data where appropriate.

Model diagnostics were performed by inspecting residual plots, including QQ plots, residuals versus fitted values, and residuals versus predictors. Cook’s distances were computed to identify influential observations. Statistical significance was evaluated at α = 0.05 throughout [37].

## 3. Results

### 3.1. Pollinator Contribution: Pods, Seeds and Seed-Set

**Figure 1:**
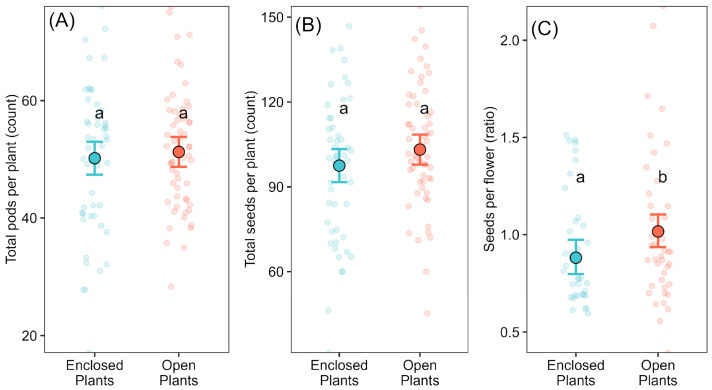
Effects of pollination treatment on soybean reproductive variables at the plant level. (**A**) Total pods per plant, (**B**) total seeds per plant, and (**C**) seed set (seeds per flower) under two treatments: Enclosed (pollinator-excluded; blue) and Open (pollinator-accessible; red). Transparent circles show raw data (jittered); filled circles with error bars indicate predicted means ± 95% confidence intervals from Gaussian models. Different letters indicate significant differences between treatments (Bonferroni-adjusted comparisons, *p* < 0.05).

The number of pods and seeds per plant did not differ significantly between pollination treatments. Enclosed plants produced 50.17 ± 1.41 pods and 97.50 ± 2.95 seeds, while open-pollinated plants produced 51.25 ± 1.28 pods and 103.17 ± 2.68 seeds (Figure 1A,B). In contrast, seed set (measured as the number of seeds per flower) was significantly higher in open-pollinated plants (1.02 ± 0.04) than in enclosed plants (0.88 ± 0.05), indicating a 15.9% increment under natural pollination conditions (Figure 1C).

All three reproductive variables (pods, seeds, and seed set) varied significantly between cultivar and season (Table 1). Among the cultivars assessed, ‘DM8002’ and ‘DM8277’ generally performed better in total pod and seed output, whereas ‘DM8277’ and ‘NS7211’ showed the highest seed set values (Table S1). Plants grown in 2018 produced fewer pods and seeds overall than those in 2017. Apparent seasonal differences in seed set values should be interpreted with caution, as they may partly reflect the use of different flower estimation methods (direct census in 2017 versus abscission scars in 2018). Importantly, the effect of pollination treatment on seed set (open > enclosed) was consistent across years, indicating that the main inference is robust to this methodological difference. The final model for the seed set explained approximately 40% of the variance, while those for pods and seeds per plant each explained around 25% (see Table 1).

### 3.2. Pollen Limitation

**Figure 2:**
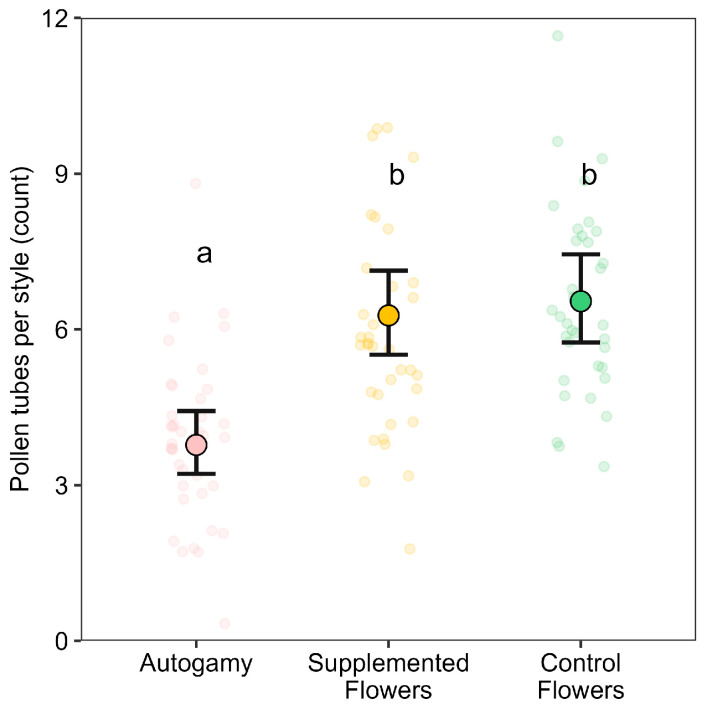
Pollen-tube growth under three pollination treatments: Autogamy (pink), Control Flowers (CF; green), and Supplemented Flowers (SF; yellow). Transparent circles represent individual flower-level data (jittered); filled circles and error bars show predicted means ± 95% confidence intervals from a Poisson model. Different Letters denote statistically significant differences among treatments (Bonferroni-adjusted comparisons, *p* < 0.05).

Supplemented and Control Flowers had significantly higher mean pollen-tube counts than the enclosed (autonomous selfing) condition (χ^2^(2) = 31.99, *p* < 0.0001), with no significant difference between them. On average, enclosed flowers had 3.78 ± 0.08 pollen tubes, compared to 6.27 ± 0.06 in supplemented flowers and 6.54 ± 0.06 in control flowers. These values represent increases of approximately 66% and 73% over the enclosed treatment, respectively (Figure 2). Calculated marginal R^2^ suggests that pollination treatment alone explained 27% of the observed variation in pollen-tube counts. Overall, these results demonstrate that both supplemental pollination and exposure to pollinators significantly increase pollen-tube growth relative to autonomous selfing conditions, highlighting the importance of pollen transfer in enhancing pollen-tube formation in soybean flowers.

**Figure 3:**
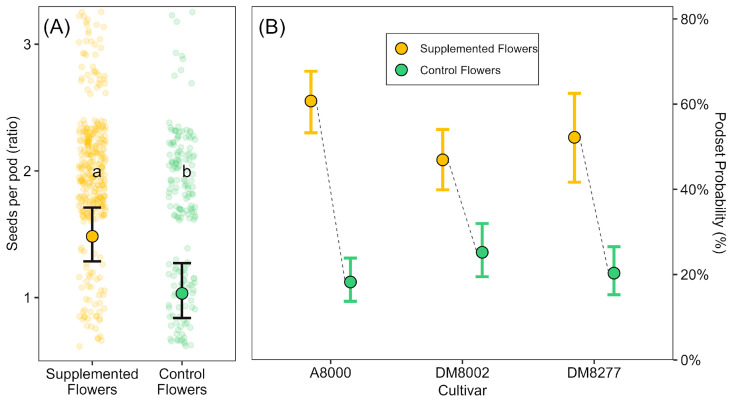
Effects of pollen supplementation on flower-level reproductive outcomes. Supplemented Flowers (yellow) and Control Flowers (green) are shown in both panels. Transparent circles indicate raw data, and filled circles with error bars show model-predicted means ± 95% confidence intervals. (**A**) Seeds per pod (excluding zero-seed pods) from the count component of a hurdle model. Different letters indicate significant differences between treatments (Bonferroni-adjusted comparisons, *p* < 0.05). (**B**) Probability of pod set across three cultivars (A8000, DM8002, DM8277) from the zero-inflation component of the same model. A significant treatment × cultivar interaction was detected.

We found that under natural pollination conditions (CF), the probability of pod set was approximately 19%, while under supplemental pollination, this likelihood increased markedly to about 61%, indicating that the SF treatment substantially enhanced pod formation. A statistically significant interaction between pollination treatment and cultivar (χ^2^(2) = 11.52, *p =* 0.003) indicated that the strength of this effect varied among cultivars. However, the direction of the response was consistent across cultivars (supplemental pollination increased pod formation in every case), though the magnitude of the effect was weaker in ‘DM8002’, suggesting that the benefit of pollen supplementation in reducing pod failure is cultivar-dependent (Figure 3B).

When focusing on flowers that successfully set a pod (conditional part of the model), both pollination treatment and cultivar significantly influenced the number of seeds per pod. We found that supplemental pollination increased seed number averaging 1.48 ± 0.07 seeds per pod, compared to 1.03 ± 0.11 in control flowers, reflecting an approximate 40% gain (Figure 3A). Among cultivars, ‘DM8277’ showed a notably higher seed number per pod relative to ‘A8000’ (approximately +38%), while ‘DM8002’ did not differ significantly from the reference. These results indicate that supplemental pollen not only increases the likelihood of pod formation but also enhances seed production within pods, with consistent effects across cultivars.

**Figure 4:**
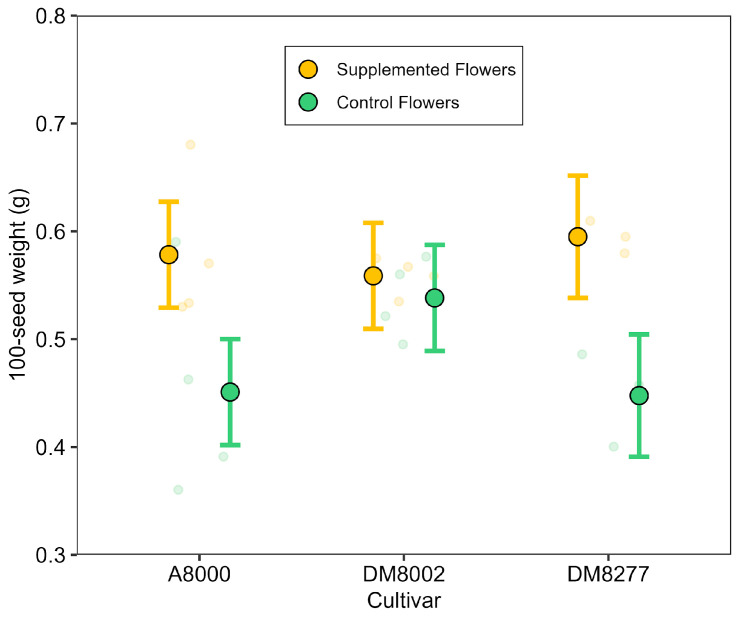
Seed weight (grams per 100 seeds) across three soybean cultivars (A8000, DM8002, DM8277) under two treatments: Supplemented Flowers (yellow) and Control Flowers (green). Transparent circles represent raw data; Treatment × cultivar interaction was significant; filled circles and error bars show model-predicted means ± 95% (Gaussian GLMM) for each combination.

Seed weight per 100 seeds was influenced by both pollination treatment and cultivar, with a significant interaction between these factors (χ^2^(2) = 6.87, *p* = 0.032). Overall, supplemental pollination increased seed weight, but the magnitude of this effect varied across cultivars. In ‘A8000’ and ‘DM8277’, supplemented flowers produced significantly heavier seeds than their respective control flowers (about 0.13–0.15 g more on average) while no significant difference was observed in ‘DM8002’ (Figure 4). These findings are consistent with the interaction term, indicating that cultivar identity modulates the effect of supplemental pollen on seed development (the final model explained 57% of the variance). Residual diagnostics indicate some deviations, likely due to the small sample size (n = 22) relative to the number of parameters estimated. Nonetheless, as a robustness check, a simpler mixed-effects model treating plant identity as a random effect confirmed the overall treatment effect (Δ = 0.094 g per 100 seeds, Supplemented > Control). Ultimately, these results suggest that supplemental pollination may enhance seed weight, although the magnitude of this effect could vary among cultivars.

## 4. Discussion

Our findings highlight the interplay between soybean’s capacity for self-pollination and its responsiveness to externally sourced pollen, either through insect visitation or manual supplementation. At the whole-plant scale, preventing insect visitation to flowers did not significantly alter total pod or seed production, although open pollination did increase seeds per flower. Manual cross-pollination markedly enhanced pod set probability and seed production within pods, revealing that pollen limitation can constrain yield when viewed on a per-flower basis. Thus, selfing may be adequate to maintain moderate yield at the plant scale; however, additional, high-quality pollen can enhance reproductive outcomes for individual flowers [24,38]. Importantly, interpreting results across these two scales is not trivial: while flower-level assessments can help reveal mechanistic insights into the potential benefits of pollen import, such as improved pod set and seed number, plant-level outcomes are more directly relevant to farmers, who are ultimately concerned with total yield per unit area. This distinction highlights the importance of bridging mechanistic understanding with agronomic relevance when assessing pollinator contributions.

Despite soybean’s predominantly autogamous mating system, we detected a ~15.9% higher seed set (seeds per flower) under open pollination, indicating greater per-flower reproductive efficiency. However, this effect did not translate into significant differences in total seeds or pods per plant, in contrast to most previous studies [16,17,39,40,41], which reported pollinator-mediated gains at the whole-plant yield level. Seasonal differences in seed set likely reflect the different flower-counting methods used, but treatment effects were consistent across years. We also note that plant-level replication was modest (5–7 plants per cultivar × treatment per year), which limits power to detect small whole-plant effects; accordingly, the plant-level null should be interpreted with caution as “no effect detected under our conditions”.

Several contextual factors may help to explain this limited pollinator contribution. Although insect visitation was confirmed by personal observations (*Apis mellifera* and halictid bees), pollinator activity was not quantified, leaving uncertainty about visitation rates and visitor identity. Our small-scale setup may also have attracted limited pollinator attention, especially given the availability of surrounding native flowering species. Moreover, unlike prior studies conducted under field conditions, our use of pots may have constrained root development and reduced the plants’ ability to translate additional pollen into greater yield. This limitation could be relevant given that soybean roots can reach depths of up to 2 m [42], and both water and nutrient acquisition are known to scale with root volume [43]. Nonetheless, because all plants experienced the same pot conditions and watering regime, treatment contrasts are still comparable. Additionally, the pot setup facilitated pollen supplementation in soybean, a crop whose small flowers are difficult to manipulate in the field, and allowed direct cultivar comparisons under uniform conditions—something rarely possible in farm settings. More broadly, these results fit a two-stage view: pollen receipt governs per-flower success, while whole-plant seed production is contingent on resource availability [44,45].

Fully supplementing every flower on plants is the ideal scenario for estimating pollen limitation at the plant scale, but generally impractical; consequently, most studies (including ours) treat a subset of flowers [44,45]. Such partial-plant designs can inflate effect sizes if resources are reallocated from untreated to treated flowers. We placed efforts to mitigate this by supplementing a large fraction of flowers per plant (mean ≈ 44% across 11 plants; 1446 flowers) and by testing for reallocation directly: increasing the supplemented fraction did not reduce seed production in unsupplemented flowers (Appendix A). While seeds per plant is the preferred agronomic metric, partial measures (e.g., fruit/seed set) are informative proxies and correlate positively with plant-level output across studies [45]. We therefore interpret the strong flower-level effects as evidence that pollination can enhance per-flower success, while recognizing that whole-plant yield responses may be constrained by resource context and by differences between hand-applied and naturally delivered pollen.

In contrast to the modest pollinator contribution mentioned earlier, our pollen supplementation experiment revealed clear evidence of pollen limitation in soybean. Pollen limitation, understood as reduced fruit and seed production due to insufficient or suboptimal pollen receipt [23], is generally considered less prominent in self-compatible hermaphroditic species like soybean [44,45]. Nevertheless, our results, along with previous studies reporting improved yields under biotic pollination [16,17,41], suggest that soybean reproduction may benefit from enhanced pollen delivery under certain conditions. Indeed, Evans et al. (2023) recently demonstrated significant improvements in soybean fruit weight due to cross-pollination and proximity to floral resources, further supporting the idea that pollen supplementation, especially from genetically diverse sources and enhanced pollinator activity, positively influences reproductive success in soybean [24].

In our experiment, supplemental pollination enhanced pod set, seed number per pod, and seed weight. Notably, cross-pollen supplementation increased the likelihood of pod set from 19% to 61%, and resulted in seed numbers per pod that were approximately 40% higher than those of unsupplemented flowers. These findings indicate that even in a predominantly self-pollinating crop like soybean, reproductive success can be limited by the quantity or quality of available pollen. Although a significant interaction between treatment and cultivar was detected, the effect of pollen supplementation was positive across all cultivars, suggesting a general responsiveness to improved pollen delivery. Variation in the strength of this response may reflect underlying differences in cultivar traits [21].

This reproductive success enhancement can be explained by a combination of mechanical and genetic factors. The process of hand pollination may have stimulated pollen growth by physically contacting the floral reproductive column, potentially enhancing both assisted selfing and cross-pollination [17]; however, because control flowers were not mock-handled, we cannot entirely exclude a contribution of mechanical stimulation to the observed effect. Moreover, the introduction of genetically distinct pollen may have enhanced ovule fertilization success and potentially mitigated the effects of inbreeding [46]. While the influence of internal resource reallocation within plants cannot be entirely disentangled, we observed no pattern suggesting that the proportion of supplemented flowers impacted seed development in unsupplemented flowers (Appendix A, Figure A1). These results are consistent with the presence of pollen limitation in soybean, particularly related to pollen quality, though further work is needed to explore this under field conditions [24].

Our previous field-based studies have shown that soybean yield responses to pollinators vary across both broad geographic gradients and local cultivar traits. In a global synthesis of 28 exclusion experiments, Cunha et al. (2023) reported that soybean dependence on pollinators averaged ~30% but decreased sharply at higher latitudes, whereas in the Chaco region of Argentina, Chacoff et al. (2024) found that pollinator contribution reached up to 40% and differed according to cultivar traits such as flower color and maturity group [21,22]. Here, by conducting controlled common garden experiments, we complement these field-scale findings with direct hand-pollination tests, demonstrating that soybean reproduction can also be limited by pollen availability and quality at the flower level. Field studies using different approaches converge on similar conclusions: adjacent pollinator habitat increased seed weight by 6.5% [47], in-field nesting substrate boosted seeds and pods by ~30% [40], soybean production increased after adding honeybees [16,17,48], and pollen deposition differed significantly under traditional pollinator exclusion versus open-pollination treatments [49]. Taken together, these findings show that pollinator activity can impact soybean production, yet the drivers of the strong context dependence across studies remain poorly understood. Moving forward, large-scale field research that integrates pollinator activity with at least some combination of cultivar choice, landscape context, and environmental factors will be crucial to determine when flower-level responsiveness can realistically translate into yield benefits at the crop scale.

The results discussed here underscore the importance of considering both the ecological and agronomic dimensions of pollination in self-compatible crops such as soybean. Although soybean is typically regarded as a largely autonomous crop, the observed improvements in seed set, pod formation, and seed weight suggest that enhanced pollen input, whether from wild pollinators or managed practices, can play a supportive role in maximizing reproductive success. Importantly, the relevance of such benefits depends on context: flower-level experiments can uncover subtle but biologically meaningful effects, whereas producers are ultimately interested in outcomes at the scale of fields and harvests. Bridging this gap requires continued efforts to link mechanistic understanding of pollination with practical, scalable recommendations for crop management, particularly as agricultural systems face growing pressures to meet market demands while advancing environmental sustainability.

## Figures and Tables

**Table 1:** Summary of fixed effects for all models evaluating soybean reproductive outcomes at plant and flower levels.

**Fixed Effects**			**Pods**		**Seeds**		**Seed Set**	
**Plant Level**			**χ^2^ (*df*)**	***p***	**χ^2^ (*df*)**	***p***	**χ^2^ (*df*)**	***p***
			R^2^ = 0.25		R^2^ = 0.25		R^2^ = 0.40	
Treatment			0.31 (1)	0.573	2.00 (1)	0.156	5.28 (1)	**0.021**
Cultivar			23.54 (3)	**<0.001**	13.09 (3)	**0.004**	14.69 (3)	**0.002**
Season			13.05 (1)	**<0.001**	18.35 (1)	**<0.001**	10.75 (1)	**0.001**
**Fixed Effects**	**Pollen Tubes**		**Seeds (Zero-Inflation)**		**Seeds (Conditional)**		**Seed Weight (×100)**	
**Flower Level**	**χ^2^ (*df*)**	***p***	**χ^2^ (*df*)**	***p***	**χ^2^ (*df*)**	***p***	**χ^2^ (*df*)**	***p***
	R^2^ = 0.27		R^2^ = 0.13				R^2^ = 0.31	
Treatment	31.98 (2)	**<0.001**	116.58 (1)	**<0.001**	13.06 (1)	**<0.001**	19.30 (1)	**<0.001**
Cultivar	—	—	0.91 (2)	0.633	10.68 (2)	**0.004**	2.00 (2)	0.367
Treatment:Cultivar	—	—	11.51 (2)	**0.003**	—	—	6.87 (2)	**0.032**

Values indicate chi-square statistics (χ^2^), degrees of freedom (*df*; numbers in parentheses), and associated *p*-values from Wald χ^2^ tests (or likelihood ratio tests for zero-inflated and conditional models). R^2^ values represent marginal R. “Seed set” refers to the average number of seeds per flower; “Seed weight” is given per 100 seeds. “Zero-inflation” and “Conditional” refer to the two components of the hurdle model applied to pod and seed production data. *p*-values in bold indicate statistical significance (*p* < 0.05).

## Data Availability

The original data presented in the study are openly available in the Repositorio Institucional CONICET Digital at http://hdl.handle.net/11336/268577 (accessed on 22 September 2025).

## Supplementary Materials

The following supporting information can be downloaded at: https://www.mdpi.com/article/10.3390/plants14192964/s1, Figure S1: Timeline of soybean experiments in 2017 (A) and 2018 (B); Figure S2: Representative microscopic images of the distal gynoecium captured through the ocular lens; Table S1: Post hoc comparisons (lsmeans) across soybean cultivars for pods per plant, seeds per plant, and seed set.

## Abbreviations

The following abbreviations are used in this manuscript:

SF	Supplemented Flowers
CF	Control Flowers
GLMM	Generalized Linear Mixed Model
LRT	Likelihood Ratio Test
MG	Maturity Group

## Appendix A

To explore whether increased seed production in supplemented flowers occurred at the expense of unsupplemented ones—i.e., via resource reallocation within the plant—we conducted a complementary analysis at the plant level. We fitted a linear regression model using the proportion of supplemented flowers per plant as a predictor of total seeds per flower. If strong within-plant resource reallocation were occurring, we would expect no overall gain in seed set per flower with increasing supplementation. Contrary to this expectation, we found a significant positive association (β = 0.23 ± 0.06 SE, *p* = 0.0001), indicating that total reproductive output per flower increased with the fraction of supplemented flowers (Figure A1). This pattern suggests that pollen limitation, rather than resource reallocation, was the primary constraint on seed set in our experimental conditions.
