# Supplementary material for: Assessment of Pollen Limitation and Pollinators’ Contribution in Soybean (Glycine max)
Source: Plants (Basel). 2025 Sep 24;14(19):2964. doi: 10.3390/plants14192964 (PMC12525767; doi:10.3390/plants14192964)
Supplement: Supplementary file 1 [file plants-14-02964-s001.zip › plants-3822885-supplementary.pdf]

Supplementary Materials

Experiment Timeline (2017)

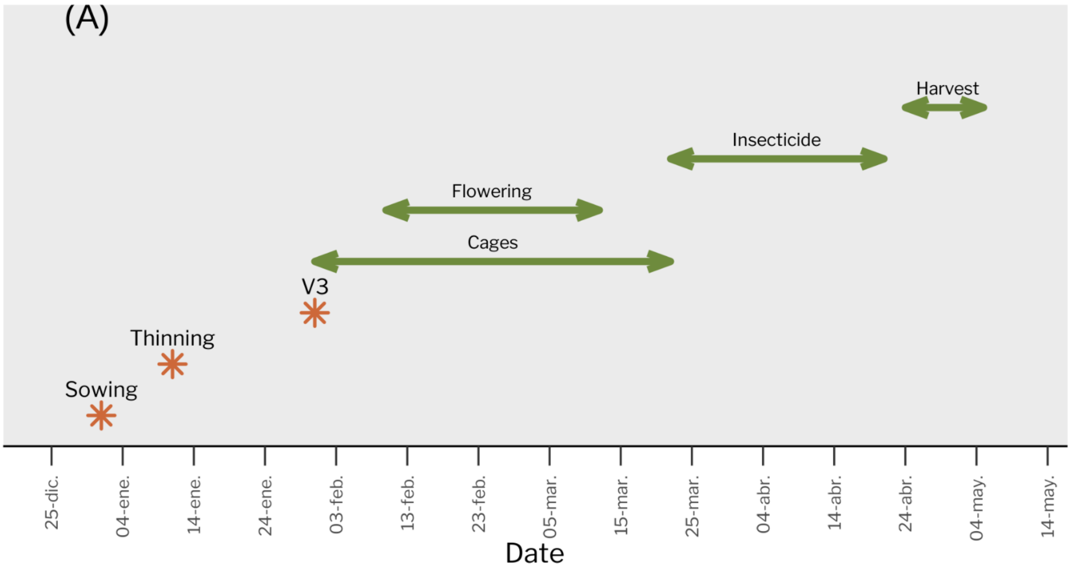

Experiment Timeline (2018)

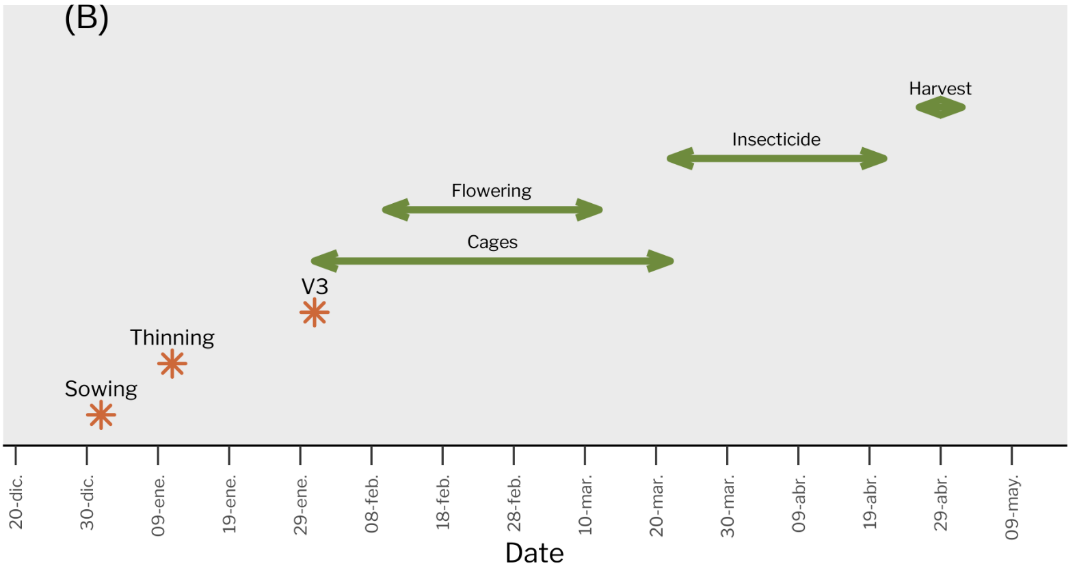

Figure S1. Timeline of soybean experiments in 2017 (A) and 2018 (B). We conducted two consecutive soybean experiments between January and May of 2017 and 2018 at the Instituto de Ecología Regional (Tucumán, Argentina). Each panel displays the schedule of key experimental events, including sowing, thinning, and V3 stage (point events), as well as activities with duration such as caging (starting date indicates the day cages were set up; and ending date is the day cages were removed), flowering period, insecticide applications, and harvest. All plants were grown outdoors in pots under full sunlight. Dates reflect actual implementation across both years.

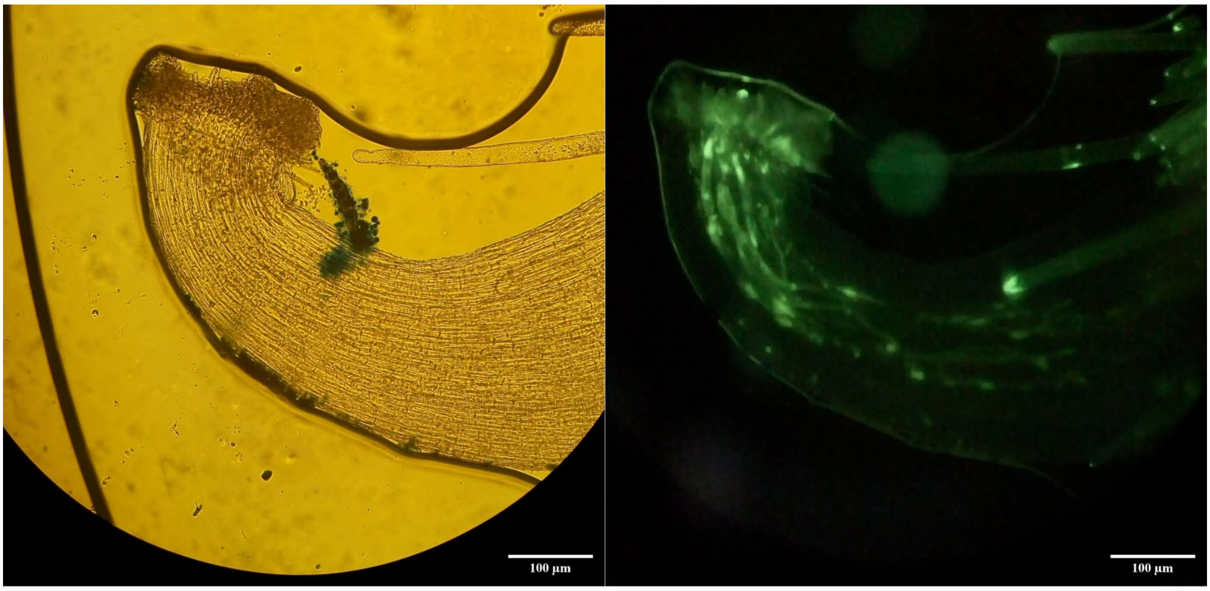

Figure S2. Representative microscopic images of the distal gynoecium captured through the ocular lens. Left: bright-field view of a distal gynoecium. Right: the same specimen under UV excitation (mercury lamp), revealing pollen tubes as bright fluorescent strands extending down the style. Pollen tube growth was assessed following the staining protocol described in the main text and was quantified by direct microscopic observation; these images are provided as illustrative examples only. Images were acquired with a Motorola C Plus smartphone. Scale bars = 100 µm (estimated from mean pollen diameter; mean pixel diameter 64.75 ± 4.64 px, n = 40; literature pollen diameter 25 µm).

Table S1. Post-hoc comparisons (lsmeans) across soybean varieties for pods per plant, seeds per plant, and seed set.

Cultivar	Pods per Plant	Seeds per Plant	Seed Set
NS7211	45.2 (2.07) a	90.8 (4.41) a	0.99 (0.06) bc
DM8277	47.6 (1.92) ab	110.1 (4.11) b	1.17 (0.09) c
A8000	53.4 (2.03) bc	95.6 (4.33) ab	0.69 (0.04) a
DM8002	56.7 (2.03) c	102.9 (4.33) ab	0.92 (0.05) b

Post-hoc comparisons (Bonferroni-adjusted) of soybean varieties across three reproductive metrics for the pollinators' contribution experiment: total pods per plant, total seeds per plant, and average seed set per flower (back-transformed from the log scale). Values represent mean (SE). Different letters within each column indicate significant differences at $\alpha = 0.05$. Results are averaged across pollination treatments.
